# An Optimized Whole-Body Cortisol Quantification Method for Assessing Stress Levels in Larval Zebrafish

**DOI:** 10.1371/journal.pone.0079406

**Published:** 2013-11-01

**Authors:** Chen-Min Yeh, Mario Glöck, Soojin Ryu

**Affiliations:** Developmental Genetics of the Nervous System, Max Planck Institute for Medical Research, Heidelberg, Germany; Tulane University Medical School, United States of America

## Abstract

Glucocorticoids serve important regulatory functions for many physiological processes and are critical mediators of the stress response. The stress response is a set of bodily processes aimed at counteracting a state of threatened homeostasis. Proper stress response is critical for the survival of an animal, however prolonged or abnormal stress response can be detrimental and is implicated in a number of human diseases such as depression and metabolic diseases. To dissect the underlying mechanism of this complex and important response, the zebrafish, *Danio rerio* offer important advantages such as ease of genetic manipulations and high-throughput behavioral analyses. However, there is a paucity of suitable methods to measure stress level in larval zebrafish. Therefore, an efficient low-cost method to monitor stress hormone levels will greatly facilitate stress research in zebrafish larvae. In this study, we optimized sample collection as well as cortisol extraction methods and developed a home-made ELISA protocol for measuring whole-body cortisol level in zebrafish larvae. Further, using our customized protocols, we characterized the response of larval zebrafish to a variety of stressors. This assay, developed for efficient cortisol quantification, will be useful for systematic and large-scale stress analyses in larval zebrafish.

## Introduction

The stress response relies heavily on the hypothalamic-pituitary-adrenocortical (HPA) axis and its pleiotropic final effector glucocorticoids (GCs) [1]. Under stressful situation, neurons in the hypothalamic paraventricular nucleus (PVN) release hormones such as corticotropin-releasing hormone (CRH) and arginine vasopressin (AVP) into the hypothalamo-pituitary portal circulation. Acting on the pituitary gland, these hormones then trigger the release of adrenocorticotropic hormone (ACTH) into the blood, which induces, in turn, the release of GCs from the adrenal cortex. GCs exert pleiotropic actions in the central nervous system and the periphery. Under both basal and stressful conditions, they serve multiple functions such as regulation of the HPA-axis activity and modification of cellular metabolism, and immune activities [2].

As a vertebrate amenable to genetic manipulation and high throughput behavioral and physiological analysis due to their small size, larval zebrafish, *Danio rerio*, offer important advantages for stress and neuroendocrine research. Recent works demonstrated functional and anatomical similarities between the zebrafish hypothalamic-pituitary-interrenal gland (HPI) axis and the HPA axis in mammals [3-5], arguing that larval zebrafish can be used for studying stress physiology and behavior [6,7]. Furthermore, it has been reported that both adult and larval zebrafish respond to stressors with increased cortisol levels and changes of gene expression [6,8-15]. Further, important aspects of GC receptor signalling are likely to be conserved between zebrafish and mammals [16-18]. Yet, despite these advances, the precise relationship between stress-related GC level and physiological and behavioral change remains largely untested. 

In zebrafish, cortisol is currently being measured using radioimmunoassay (RIA) and ELISA. RIA protocols optimized for larval zebrafish have recently been reported [9], but ELISAs so far rely on commercial kits. Hence, at a price of around two hundred dollars per plate, extensive, large-scale characterization of the stress response is only possible at prohibitive costs. Also, most commercial cortisol kits have been optimized for measuring cortisol from body fluid and vary in their efficiency and sensitivity, possibly due to differences in reagents and techniques [19]. To overcome these limitations, we optimized sample collection as well as cortisol extraction procedures and developed a home-made ELISA protocol specifically for measuring whole-body cortisol in developing zebrafish larvae. Using these protocols, we tested a variety of stressors and showed how diverse environmental stimuli can lead to a cortisol increase in larval zebrafish. Thus, our work allows for routine usage of a standardized cortisol quantification method in larval zebrafish, which are particularly well-suited for analyses of the effects of early life stress as well as the ontogeny of the stress response. 

## Materials and Methods

### Ethics Statement

All experiments performed in this study were conducted according to the guidelines of the German animal welfare law and were approved by the local government (Regional Council Karlsruhe) under the protocol 35-9185.81/G-29/12.

### Zebrafish husbandry

Zebrafish (cross between AB and TL strains) were maintained and bred under standard conditions at 28.5°C with 12/12 light dark cycle [20]. For determining the optimum number of larvae to be raised, 10, 30, 45 or 60 embryos per well were raised in a 6-well plate with 3.5 cm inner diameter containing 5 ml E2 medium. Larval length was measured from the anterior tip of the larval head to the posterior end of the caudal fin at 2 days post fertilization (dpf) evening. For all the other experiments, between 31-33 zebrafish embryos per well were raised until 5 dpf. 

### Cortisol Extraction and ELISA

Larvae were immobilized in ice-cold water and a group of 30 larvae were collected as one sample. After excess water has been removed, the samples were frozen in ethanol (EtOH)/dry-ice bath. Homogenization of the samples was performed with a pellet mixer (VWR International) for 20 seconds. Ethyl acetate was added to homogenate, the supernatant was collected and vaporized. Cortisol was dissolved in 0.2% Bovine serum albumin (A7030, Sigma) in phosphate-buffered saline (PBS) and frozen. 

For cortisol ELISA, the minimum cortisol antibody coating time was first determined by the signal-to-noise ratio calculated from wells coated for 1, 3, 6, 16, 24 or 40 hours. For all the other cortisol ELISA experiments, 96-well plates (VWR International) were coated for 16 hours at 4°C with cortisol antibody (P01-92-94M-P, EastCoast Bio) solution (1.6 g/mL in PBS), washed and blocked with 0.1% BSA in PBS. Cortisol samples and cortisol-HRP (P91-92-91H, EastCoast Bio) were incubated at room temperature for 2 hours and washed extensively with PBS containing 0.05% Tween-20 (Roth). Color reactions were performed using Tetramethylbenzidine (TMB:22166-1, Biomol) and Tetrabutylammonium borohydride (TBABH: 230170-10G, Sigma) and stopped using 1M H_2_SO_4_. For calculating the pH-dependency of the color reaction, diluted HRP with TMB substrate was incubated for 10 minutes at room temperature at different pH levels and read immediately after ending the reaction. Absorbance at 450 nm was read in ELISA plate reader (Multiskan Ascent Microplate Photometer, Thermo Scientific). Comparisons with commercial kits were carried out using Cortisol ELISA Kit (RE52611, IBL International). A detailed step-by-step protocol (Table S1) as well as recipes for all the solutions and stock reagents for the cortisol extraction and ELISA (Table S2) are provided.

### Stressor Treatments

For chemical, osmotic and acidic shocks, 1 ml of either 5x concentrated medium (250 mg ml^-1^ Ammonia solution (#6774, Roth); 10% EtOH; 250 µM CuSO_4_; 1.25 M NaCl; 5 mM HCl) or control medium (prewarmed to 28°C) was added to each of the wells of the 6-well-plates containing larvae in 4 ml of medium. The larvae were incubated for 10 minutes, immobilized in ice-water and collected immediately afterwards. For temperature shock, the larvae were incubated in a water bath at either 36°C or 16°C for 10 minutes and then collected. 

### Statistical analysis

**Figure 1:**
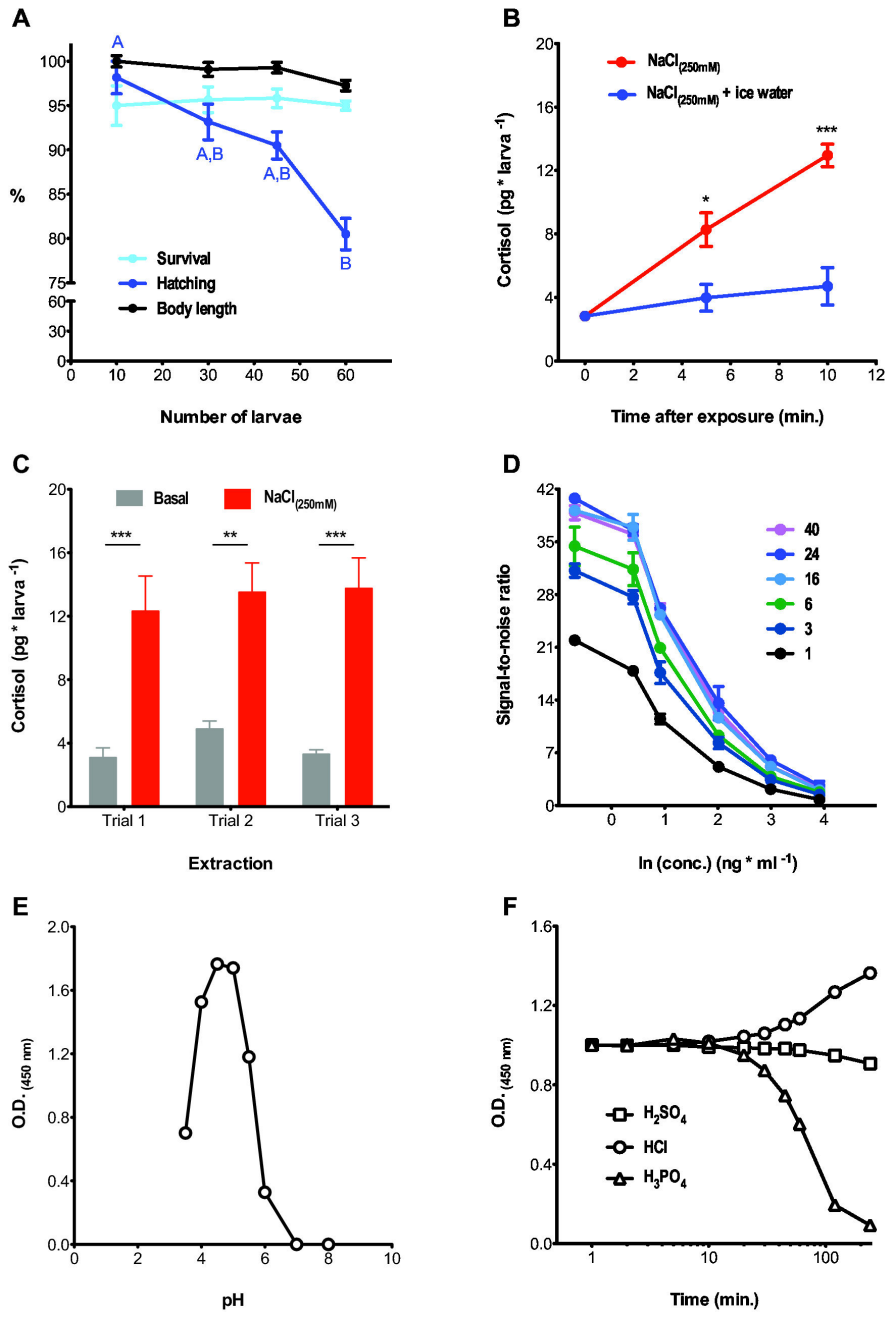
Optimization of sample collection, extraction and ELISA protocols to measure cortisol level in zebrafish larvae. (**A**) The relationship between the number of larvae per raising well and survival (light blue circles, in %; Kruskal-Wallis test, *H*=1.0, p = 0.80, N = 6), hatching (dark blue circles, in %; Kruskal-Wallis test, *H*=15.6, p < 0.01, N = 6; uppercase letters depict differences between groups, Dunn's multiple comparison test for pair comparisons,) and body length (black circles, in %; Kruskal-Wallis test, *H*=8.3, p = 0.04, N = 15; no pair differences detected by Dunn's multiple comparison test for pair comparisons) at 2 dpf evening. (**B**) Exposure to NaCl (red circles) increases whole-body cortisol in 5 dpf larvae. Submerging the larvae in ice-cold water immediately after NaCl exposure (blue circles) efficiently prevents the raise of cortisol caused by the osmotic shock (red vs. blue circles, Kruskal-Wallis test, osmotic shock–red circles: *H*=17.1, p < 0.001, osmotic shock followed by ice-cold water incubation–blue circles: *H*=1.5, p = 0.47). For each exposure time, asterisks designate statistical differences between pairs, *p < 0.05, ***p < 0.001 (Two sample t-tests, non-exposed: t_(12)_ = 0.0, p = 1.0, 5 min.: t_(11)_ = 3.1, p = 0.01, 10 min.: t_(12)_ = 6.0, p < 0.0001). (**C**) Cortisol level in NaCl-exposed and non-exposed larvae remain the same across experiments and independent extractions (Two way ANOVA, treatment: F_(1,18)_ = 62.9, p < 0.001; extraction: F_(1,18)_ = 0.53, p = 0.60; treatment x extraction: F_(1,18)_ = 0.20, p = 0.82; Bonferroni’s post-tests, **p < 0.01 and ***p < 0.001 for comparisons of pairs across extractions). (**D**) Signal-to-noise ratios from different cortisol concentrations (0.1-50 ng cortisol ml^-1^) in wells coated with cortisol mAB for 1-40 hours (sample size per group = 2). (**E**) An incubation of HRP with TMB at a pH level of 4.5 gives the highest signal intensity, whereas signal detection decreases sharply at pH levels lower and higher than 3.5 and 6, respectively. (**F**) Time course of signal intensity after applying different 1 M stop solutions. Among them, H_2_SO_4_ preserves signal stability overtime.

**Figure 2:**
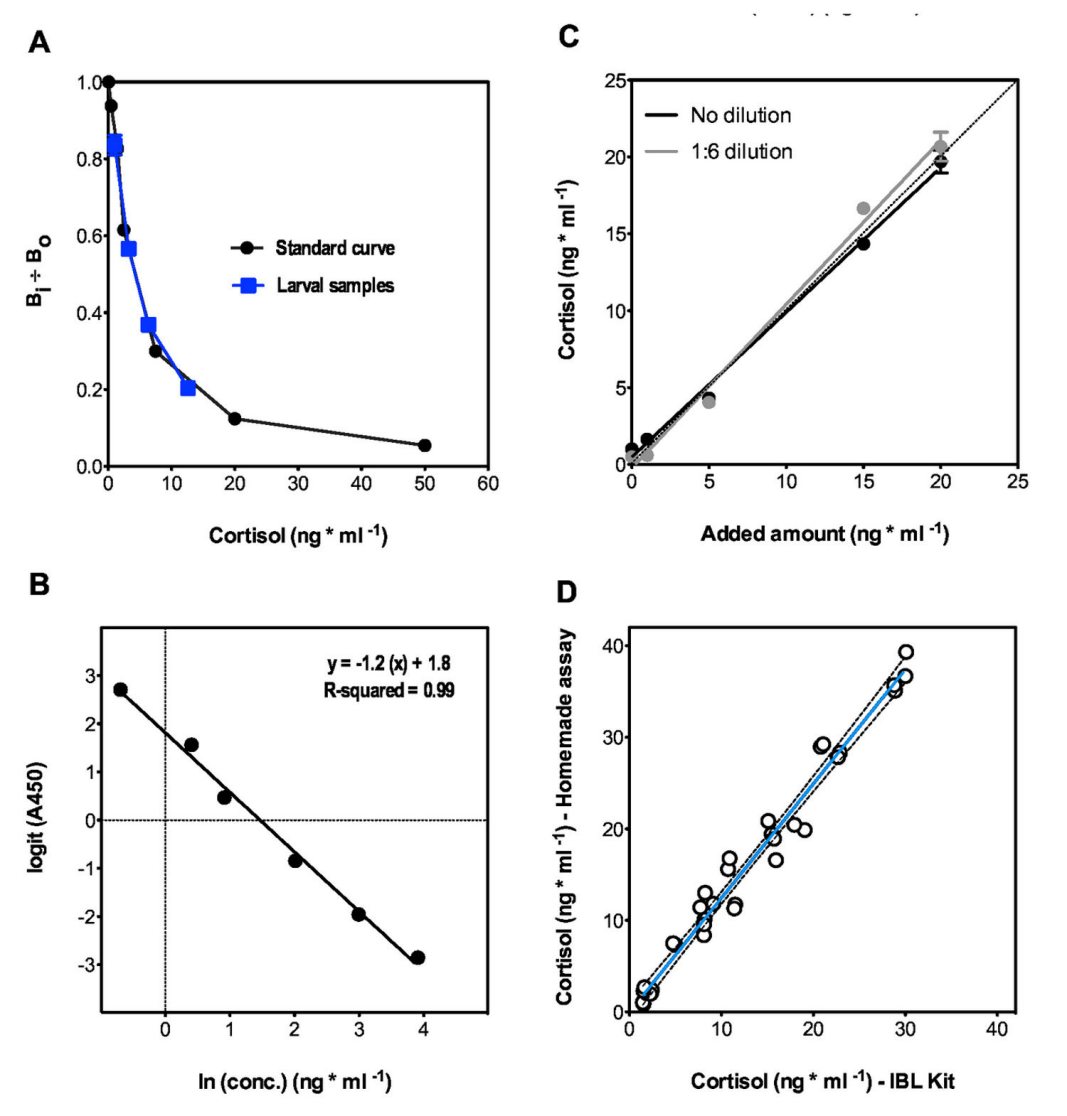
Validation of the home-made ELISA. (**A**) Standard curve of cortisol ELISA generated by plotting cortisol concentrations ranging from 0 to 50 ng cortisol ml^-1^ (♦) to the percentage bound, Bi/Bo. (Bi: the O.D. of different standards. Bo: the O.D. of maximum binding coming from standard with 0 ng cortisol ml^-1^). Serial dilutions of zebrafish sample spiked with 10 ng cortisol ml^-1^ () plotted in parallel to the standard curve. (**B**) Logit-log plot of the standard curve (Linear regression, p < 0.001, R-squared: 0.99). (**C**) Recovery functions calculated from linear regressions (p < 0.001) of samples spiked with (0/1/5/15/20) ng cortisol ml^-1^ was y = 0.9 x + 0.5 (N=6) and y = 1.1 x - 0.2 for 1:6 diluted samples (N=6). The proportional systematic errors were 0.93 and 1.07 when the samples were not diluted or diluted six times, respectively. (**D**) We compared the performance of our cortisol ELISA and that of a commercial ELISA Kit by simultaneously testing samples using both methods. The data sets from both assays correlated significantly with one another (Spearman r = 0.97, p < 0.001).

All group data are presented as mean ± standard error of the mean (S.E.M.). Two-group comparisons were made using Student’s t tests, with the null hypothesis being rejected at the *p < 0.05, **p < 0.01 or ***p < 0.001 level. Multiple group comparisons were assessed using one-way or two-way ANOVAs, followed by Bonferroni’s post-hoc test when necessary; the null hypothesis was rejected at the *p < 0.05 level. If the data did not meet the assumptions of the one-way ANOVA, we used the nonparametric Kruskal-Wallis test, followed by a Dunn’s multiple comparison test, with the null hypotheses rejected at the *p < 0.05 level. We used linear regressions and Pearson’s and Spearman’s rank correlation coefficients to compare data from Figures 1A and 2B-D. 

## Results

### Development of extraction and sample collection protocols

In order to develop a rapid, sensitive and cost-effective protocol to measure cortisol level in zebrafish larvae, we sought to optimize major steps of the procedure, from the collection of larvae, homogenization of tissues and extraction of cortisol to the ELISA procedure itself. Because crowding raises cortisol levels in adult zebrafish [21], we first determined the optimum number of larvae to be raised in multiple wells. We observed that the percentage of survival in our wells was largely invariant to group size and generally high (~90 %) (Figure 1A, light blue circles). Yet, the percentage of hatching and the body length of larvae decreased as the number of larvae per well increased (Figure 1A, dark blue and black circles). Therefore, we decided to use 30 larvae per well. Previous studies using commercial kits used diethyl ether for cortisol extraction and achieved about 75% extraction efficiency from whole larval homogenates [21]. To improve extraction efficiency, we initially tested 11 different solvents and subsequently focused on the 4 most promising solvents: butanol, diethyl ether, ethyl acetate and hexane. Among these, ethyl acetate gave the best extraction efficiency of 90 ± 3 % and partition coefficient (Table S3). Ethyl acetate extraction efficiency was measured by comparing samples containing 5 ng of cortisol ml^-1^ before and after the extraction. To study the dynamics of stress-related cortisol change, it is important to prevent cortisol changes during sample collection. It has been reported that in teleosts the application of anesthetics can affect cortisol level [22,23]. We observed that immersion in ice-cold water was sufficient to immobilize the larvae and prevent further increase in cortisol level (Figure 1B, red vs. blue circles). The stress-induced increases in cortisol level from three separate extraction procedures were comparable but the basal cortisol level varied more (Figure 1C). The inter-extraction coefficients of variation (CV) from three independent extractions from stressed and non-stressed larvae were 26.1% and 5.8%, respectively (Figure 1C).

### Development of an optimized ELISA protocol for quantifying cortisol level in zebrafish larvae

We optimized crucial parameters for the ELISA protocol, including the duration of antibody coating, pH of the staining reaction and identifying stop solution that will ensure color stability. Although one-hour incubation was sufficient to detect concentration-dependent color change, the maximum signal-to-noise ratio was apparent after an antibody incubation period of 16 hours (Figure 1D). Next, we determined the pH-dependency of the color reaction and found that a pH level of 4.5 gave the maximum absorbance (Figure 1E). To guarantee proper signal detection after ending the reaction, we also tested different stop solutions, including 1M H_2_SO_4_, HCl, and H_3_PO_4_, at either high O.D. or low O.D. values. Signal intensity did not change dramatically (<5%) during the first 20 minutes at high O.D. values (data not shown). However, at low O.D. values, only those reactions stopped using H_2_SO_4_ maintained color stability (Figure 1F). Further we optimized other parameters such as blocking reagent and washing buffer composition (data not shown).

### Validation of the home-made ELISA

Serial dilutions of larval zebrafish samples spiked with 10 ng cortisol ml^-1^ plotted parallel to a standard dose-response curve that covered a range of 0.5-50 ng cortisol ml^-1^ fell well within the linear portion of the reference line (Figure 2A). The ensuing relationship could be approximated by a linear regression that gave an R-squared value of 0.99 (Figure 2B). The sensitivity of the assay was 0.27 ng cortisol ml^-1^. Cross-reactivity was calculated by dividing the 50% binding concentration of the cortisol standard curve by the 50% binding concentration of the cross reactant. Cross reactivity values for corticosterone, progesterone, 17-OH-progesterone, 11-deoxycortisol and cortisone were 21.9%, 11.5%, 24.5%, 7.7% and 0.4%, respectively. Cortisol recovery values (in %) for samples spiked with 1, 5, or 20 ng cortisol ml^-1^ were 82%, 86% and 94%, respectively. Since we measured cortisol values in whole larval homogenates which may contain substances that may interfere with the assay, we determined the recovery function. The recovery functions representing the proportional systematic errors were 0.93 and 1.07 when the samples were not diluted at all or diluted six times, respectively (Figure 2C). We calculated the intra- and inter-assay coefficient of variation (in %) from samples spiked with 1, 5 or 15 ng cortisol ml^-1^ as estimates of the precision and reproducibility of the assay, respectively. Precision and reproducibility values were 11.0, 11.8, and 5.6% (precision) and 10.9, 5.1, and 6.0% (reproducibility) for samples spiked with 1, 5 or 15 ng cortisol ml^-1^, respectively. For comparison with a commercial kit, 34 duplicate samples were tested simultaneously using both methods. Some samples were spiked with cortisol to obtain an even distribution of the cortisol levels in the working range, and a logit-log plot was used to convert the absorbance into cortisol concentration. Also, we applied a correction factor f= 0.93 arising from the recovery function. Cortisol values obtained using our custom cortisol ELISA and a commercial ELISA Kit correlated significantly with one another (Figure 2D). 

### Using a custom cortisol ELISA to test different stressors in larval zebrafish

**Figure 3:**
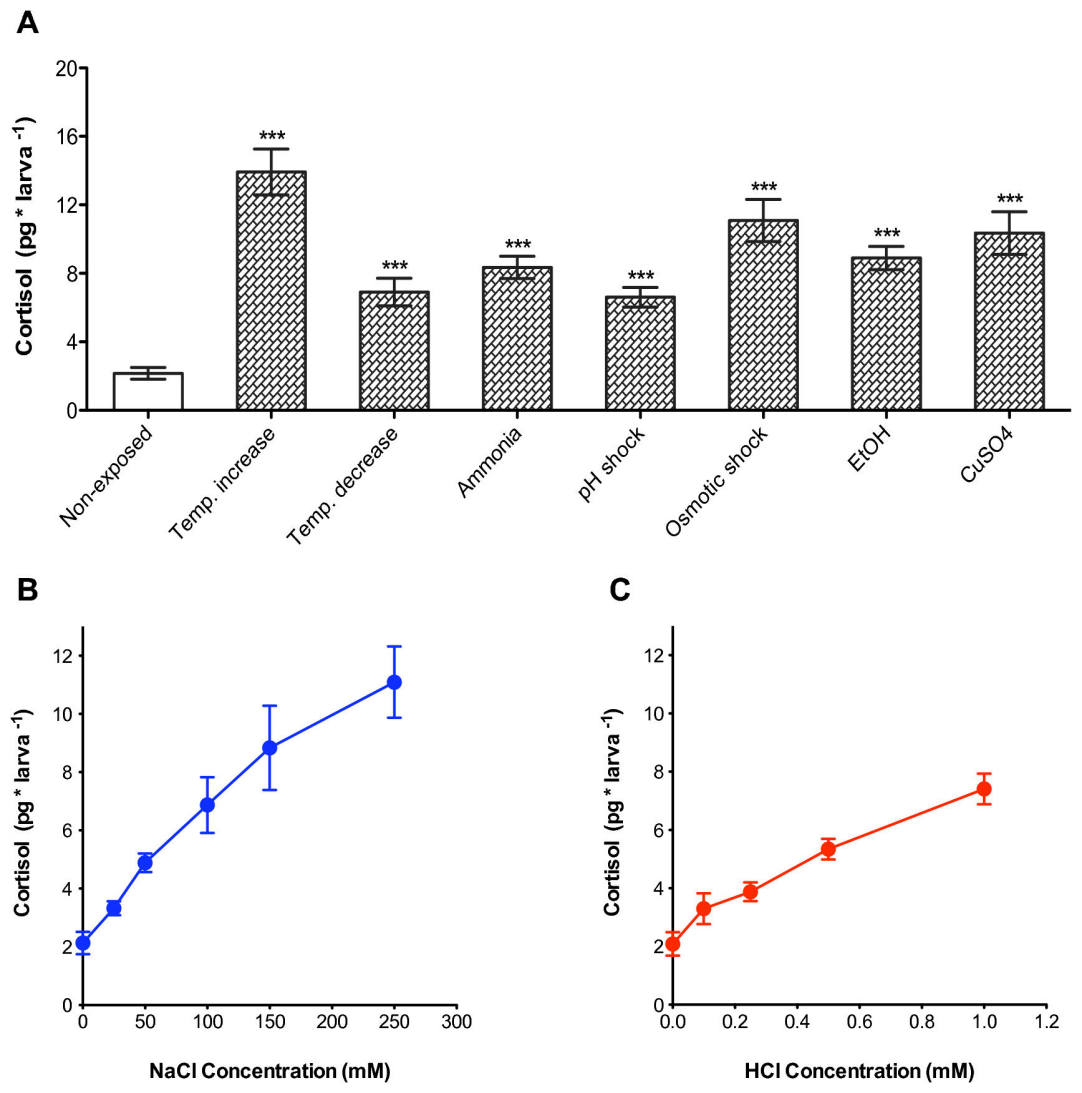
Stress-induced cortisol responses in 5 dpf larvae. (**A**) Cortisol response of 5 dpf zebrafish larvae to various stressors: non-exposed controls (N=11), temperature increase (N=8), temperature decrease (N=8), ammonia solution (50 mg ml^-1^; N=11), pH shock (1 mM; N=10), osmotic shock (250 mM; N=10), EtOH (2%; N=11), and CuSO_4_ (50 µM; N=12). The larvae responded to the various stressors with increased, varying cortisol levels (***p < 0.001 for pair comparisons of single groups against non-exposed, t test). (**B**) Cortisol dose response curve from larvae treated with 25, 50, 100, 150 and 250 mM NaCl solution (One way ANOVA, osmotic shock: F_(5,59)_ = 14.4, p < 0.001, N per group = 10). (**C**) Cortisol dose response curve from larvae treated with 0.1, 0.25, 0.5 and 1 mM HCl solution (One way ANOVA, pH shock: F_(4,44)_ = 22.3, p < 0.001, N per group = 9).

Compared to the vast amount of literature on stressors for diverse fish species [24], there is comparatively less information on stressors affecting zebrafish larvae. Although certain stimuli were reported to increase cortisol level in larval zebrafish, including osmotic and electric shocks as well as physical stressors such as swirling [9,15,25], a wide range of stressors remain untested. Therefore using our custom cortisol ELISA, we tested stress-related cortisol changes in response to various stressors in 5 dpf larval zebrafish (Figure 3A). Cortisol concentration was measured 10 minutes after the onset of stressor exposure since similar cortisol peak time has been observed [10,26]. The stressors were chosen due to their importance for general fish health and their known effects on eliciting cortisol changes [24,27,28]. Consistent with observations from earlier studies, larval zebrafish responded with increased cortisol to changes in osmolality. We observed that they also responded with increase in cortisol to changes in environmental conditions such as temperature increase, temperature decrease or lower pH. Also larval zebrafish responded robustly to the presence of toxic chemicals such as Ammonia solution, EtOH and CuSO_4_. Further, using osmotic or pH shocks, we observed dose-dependent cortisol changes indicating that our method can be used to detect subtle differences in cortisol responses (Figure 3B and C). Taken together, our results show that cortisol is a general effector of an overall response to sudden environmental changes in larval zebrafish at 5 dpf. Further at 5 dpf larvae can adjust the magnitude of their cortisol response according to the intensity of various stressful stimuli.

## Discussion

 Here we present a protocol to measure cortisol concentrations in developing zebrafish larvae. Commercial kits provide ready-to-use reagents, but they are expensive and have been optimized for measuring cortisol level in body fluids. We developed a home-made cortisol ELISA with approximately one tenth of a total cost of a commercial kit and optimized protocols for sample collection and cortisol extraction from whole larval homogenates. 

 Because our protocols used whole-body homogenates, concentration values relate not only to cortisol levels in plasma, but also to cortisol levels in various tissues. Circulating cortisol levels are generally used to determine stress levels of an individual fish. However whole-body cortisol levels have also been used in many studies to reliably determine stress levels in smaller size fish [29,30]. To correct for compounding effects of using whole larval homogenates, all our cortisol values are adjusted using extraction efficiency and recovery function to correct for matrix effects. One important issue concerning the validation of the assay is the cross-reactivity to related compounds. In this context, 21.9% cross reactivity to corticosterone is likely to be non-relevant since cortisol is the main GC regulating stress response in teleosts. Cross-reactivity to progesterone, 17-OH-progesterone and 11-deoxycortisol was also higher than values reported from the commercial kits. We therefore compared the performance of the IBL cortisol ELISA kit and our homemade assay using samples from non-stressed larvae (baseline levels) as well as samples from larvae exposed to three different stressors, namely, EtOH, HCl and NaCl. Further, we exposed the larvae to varying stressor intensity, using a 10 min. exposure to NaCl (100 mM or 250 mM) or HCl (0.5 mM or 1 mM). IBL cortisol ELISA and our cortisol ELISA gave comparable results (Pearson correlation, p < 0.01, R square: 0.90). This means that the variability associated with one of these protocols accounts for 90% of the variability associated with the other. It also indicates that our homemade ELISA provides a robust test to detect changes in cortisol level in larval zebrafish despite higher cross-reactivity to other steroids. 

 Comparing zebrafish cortisol values in different studies is not straightforward because raising conditions and measuring procedures vary widely. Also, different units have been used to report cortisol concentrations in zebrafish relating to weight, protein amount or per fish [4,21,26]. Weighing larval zebrafish itself is difficult due to their small size. It also adds a considerable amount of additional work to the general procedure. To test whether we can use protein content as a measure to correlate with cortisol level, we compared protein content and weight at different stages. In larval zebrafish, we observed that protein content per weight fish varied with developmental stage (e.g. 0.67 ng protein per mg fish at 3 dpf vs. 1.8 ng at 5 dpf and 4.3 ng at 12 dpf). Therefore it is not helpful to relate cortisol level to protein content in developing zebrafish larvae. Our assay works with groups of 30 larvae per replicate, which minimizes differences in weight or protein content across samples. These reasons made us opt for relating cortisol level to the number of larvae used per replicate, as it has been done in other studies [26]. Nevertheless, comparing and interpreting results from different sources should be done with extra care since the differences in environmental conditions and handling easily reflect on cortisol levels.

Using our home-made ELISA, we tested cortisol changes induced by various stressors. Although chemical stressors have profound impact on the health of fish, there is little information on the cortisol changes induced by high environmental copper or ammonia in zebrafish larvae. Copper treatment induced apoptosis of neuromasts as well as inflammation [31,32]. Further, high external ammonia increases the level of ammonia and urea transporters in zebrafish larvae [33]. Under these conditions, cortisol increase may aid in re-establishing the copper and ammonia homeostasis [33-35]. In adult zebrafish, EtOH exposure alters camouflage and anxiety-related behaviors and disrupts the CNS development and function in larval zebrafish [36-40]. While the role of cortisol increase by EtOH stimulation is not well understood, it is thought that EtOH suppresses the negative feedback regulation of the HPA axis and therefore, enhance the level of cortisol [41].

In conclusion, we developed a robust and low cost method for measuring whole-body cortisol level necessary for large-scale and routine quantification of cortisol level in larval zebrafish. Further, we have shown how diverse environmental stimuli can lead to a dose-dependent cortisol increase in larval zebrafish, confirming the role of cortisol as a general effector of an overall response to sudden environmental changes during larval development. Therefore, our work provides an opportunity for systematic analyses of the relationship between environmental variation and stress axis activity during development.

## Supporting Information

Table S1 **Step-by-step protocol for sample handling, cortisol extraction and cortisol ELISA.**
(DOCX)

Table S2 **Recipe for preparations of stock solutions for cortisol ELISA.**
(DOCX)

Table S3 **Extraction efficiency and partition coefficient.** Ethyl acetate shows highest partition coefficient (**P**) and extraction efficiency (**E**), compared to other solvents. C_c_: cortisol concentration; *n*_T_=2.
(DOCX)
