# Supplementary material for: An Optimized Whole-Body Cortisol Quantification Method for Assessing Stress Levels in Larval Zebrafish
Source: PLoS One. 2013 Nov 1;8(11):e79406. doi: 10.1371/journal.pone.0079406 (PMC3815139; doi:10.1371/journal.pone.0079406)
Supplement: Table S1 — Step-by-step protocol for sample handling, cortisol extraction and cortisol ELISA. (DOCX) [file pone.0079406.s001.docx]

**Supplemental Table 1**

**Larvae raising**

- Raise exactly 31-33 zebrafish embryos/well in a 6-well plate (or petri dishes with inner diameter: 3.5cm) with 5 ml E2 (or egg water) for 5 days (or other appropriate time) at 28°C and exchange medium once after hatching at 2 dpf.

**Sample collection**

- After treatments, completely fill each well of the 6-well plate with ice-water.
- Take up half of the volume in each well (without sucking up fish) and fill the well again with ice-water.
- Collect exactly 30 larvae in ice-water and transfer to prechilled 1.5 ml tube
- Spin down the tubes for 5 sec. and transfer them back on ice.
- Remove excess water (<20 µl left), freeze the samples in ethanol/dry-ice bath.
- Store samples at -20°C.

**Cortisol extraction from larvae**

- Thaw the tubes completely and add 150 µl H_2_O into them.
- Homogenize the samples for 20 sec. with a pellet mixer (VWR International).
- Add 1000 µl ethyl acetate (Sigma) to each well, vortex 30 sec. at maximum speed.
- Separate solvent and aqueous phase by centrifuging the tubes for 5 minutes at 3000x g at 4°C.
- Freeze aqueous layer on ethanol/dry-ice bath.
- Decant solvent layer into a new 1.5 ml tube.
- Evaporate solvent for 30 min. at 30°C in speed-vacuum concentrator.
- Dissolve cortisol in 60 µl Sample-Buffer (0.2% BSA in PBS) and freeze at -20°C.
- Upon use: thaw and vortex 5 min. at 1200 rpm on thermomixer at 37°C.
- Spin down for 5 sec. and use 50 µL for ELISA.

**Cortisol ELISA**

**1. Coating**

- Add 100 μl cortisol mAB-Solution (1.6 µg mL^-1^ in 1x PBS, 1:25 dilution from stock solution) into each well of the 96-Well-Plate (VWR International, 96-well Immulon 2 HB).
- Incubate at 4°C for 16 hours without shaking.
- Wash 3x with 300 µl 1x PBS-T (0.05% tween-20 in 1xPBS ).

**2. Blocking**

- Add 300 μl 1x blocking buffer (0.1% BSA in PBS) into each well and incubate at RT for 30 min.
- Wash 3x with 300 μl 1x PBS-T.

**3. Detection**

- Add 50 μl Standard, Control and Sample to respective wells of the microtiter plate (Standards: 0, 0.5, 1, 2.5, 7.5, 20, 50 ng cortisol ml^-1^ ).
- Add 50 μl Cortisol-HRP (1:80 dilution from stock in PBS) into each well of the microtiter plate.
- Incubate 2 hours at RT on an orbital shaker (40-50 rpm).
- Discard incubation solution.
- Wash 3x with 300 μl 1x PBS-T.
- Add 100µl Staining-Solution into each well (200 µL solution A to 8 ml solution B; prepare 15-30 min. before use).
- Incubate 20 min. at RT on an orbital shaker (40-50 rpm).
- Stop reaction by adding 100 µl stop-solution into each well.
- Shake briefly and read absorbance at 450 nm in ELISA plate reader (Multiskan Ascent Microplate Photometer, Thermo Scientific).
