# Supplementary material for: An Optimized Whole-Body Cortisol Quantification Method for Assessing Stress Levels in Larval Zebrafish
Source: PLoS One. 2013 Nov 1;8(11):e79406. doi: 10.1371/journal.pone.0079406 (PMC3815139; doi:10.1371/journal.pone.0079406)
Supplement: Table S2 — Recipe for preparations of stock solutions for cortisol ELISA. (DOCX) [file pone.0079406.s002.docx]

**Supplemental Table 2**

| **Chemical/Solution Name** | **Recipe for Stock Solution Preparation** |
| --- | --- |
| Anti-Cortisol monoclonal antibody  (cortisol mAB; P01-92-94M-P, EastCoast Bio) | 40 μg Cortisol mAB ml^-1^ in 1x PBS and store at -20°C. (Dilute to 1.6 µg ml^-1^ upon use) |
| Cortisol-HRP  (P91-92-91H, EastCoast Bio) | Add 1x PBS to reach total volume of 20 ml and store at 4°C. |
| Cortisol standards  (H0888, Sigma) | Dissolved 1 mg cortisol in 1 ml EtOH and add 19 ml 1x PBS to make 50 μg Cortisol ml^-1^ stock. Store at -20°C. |
| Staining solution A  (TMB, 22166-1, Biomol GmbH; TBABH, 230170-10G, Sigma) | 41 mM TMB and 8 mM TBABH in DMA. Store at 2-8°C and protected from light up to 1 year |
| Staining solution B | 3.14 μl 30% H_2_O_2_ in 10 ml Citrate-Buffer (205 mM, pH = 4,5) and store at 2-8°C, protected from light for up to 1 month. |
| Stop solution | 1M sulfuric acid |
| Embryo-Medium 2 (E2), modified | 5 ml E2-A, 1 ml E2-B, 1 ml E2-C into 950 ml milli-Q-H2O. pH was adjusted to 7,0 and 300 μL 1 % (w/v) Methylene Blue was added. Make final volume to 1 liter. |
| E2-A | 14.61 g NaCl, 0.933 g KCl, 6.163 g MgSO_4_ × 7 H_2_O, 1.02 g KH_2_PO_4_, 0.355 g Na_2_HPO_4_ and add milli-Q-H_2_O to 250 ml. Make 5 ml aliquots, store at -20°C |
| E2-B | 3.675 g CaCl_2_ and add milli-Q-H_2_O to 50 ml make 1 ml aliquots, store at -20°C |
| E2-C | 2.99 g NaHCO_3_ add milli-Q-H_2_O to 50 ml make 1 ml aliquots, store at -20°C |
