# Supplementary material for: An Optimized Whole-Body Cortisol Quantification Method for Assessing Stress Levels in Larval Zebrafish
Source: PLoS One. 2013 Nov 1;8(11):e79406. doi: 10.1371/journal.pone.0079406 (PMC3815139; doi:10.1371/journal.pone.0079406)
Supplement: Table S3 — Extraction efficiency and partition coefficient. Ethyl acetate shows highest partition coefficient (P) and extraction efficiency (E), compared to other solvents. Cc: cortisol concentration; n T=2. (DOCX) [file pone.0079406.s003.docx]

Supplemental Table 3

| **Solvent** | ***C*_C_ [ng/mL]** | **P** | **E [%]** |
| --- | --- | --- | --- |
| **Butanol** |  | 1.7 | 63.4 |
| **Extract** | 368.5 |  |  |
| **Raffinate** | 213.1 |  |  |
| **Diethyl ether** |  | 2.2 | 69.0 |
| **Extract** | 313.0 |  |  |
| **Raffinate** | 140.7 |  |  |
| **Ethyl acetate** |  | 15.2 | 93.8 |
| **Extract** | 411.2 |  |  |
| **Raffinate** | 27.1 |  |  |
| **Hexane** |  | 0.026 | 2.5 |
| **Extract** | 8.7 |  |  |
| **Raffinate** | 340.5 |  |  |
